# Natriuretic peptides and Forkhead O transcription factors act in a cooperative manner to promote cardiomyocyte cell cycle re-entry in the postnatal mouse heart

**DOI:** 10.1186/s12861-020-00236-y

**Published:** 2021-02-03

**Authors:** Mir Ali, Daniela Liccardo, Tongtong Cao, Ying Tian

**Affiliations:** Department of Pharmacology, Center for Translational Medicine, Temple University School of Medicine, Philadelphia, PA 19140 USA

**Keywords:** Neonatal heart, Natriuretic peptide signaling, ANP, BNP, FOXO, Cardiomyocyte, Cell cycle activity

## Abstract

**Background:**

Cardiomyocytes proliferate rapidly during fetal life but lose their ability of proliferation soon after birth. However, before terminal withdrawal from the cell cycle, cardiomyocytes undergo another round of cell cycle during early postnatal life in mice. While a transient wave of increased DNA synthesis in cardiomyocyte has been observed in postnatal mouse hearts, the molecular mechanisms describing cardiomyocyte cell cycle re-entry remain poorly understood. Atrial and B-type natriuretic peptides (ANP and BNP) are abundantly expressed in embryonic heart ventricles. After birth, the expression of both genes is strongly reduced in the ventricular myocardium. Forkhead O (FOXO) transcription factors are expressed in both embryonic and postnatal heart ventricles. Their transcriptional activity negatively affects cardiomyocyte proliferation. Upon phosphorylation, FOXO is translocated to the cytoplasm and is transcriptionally inactive. Despite these important findings, it remains largely unknown whether natriuretic peptides and FOXO cooperatively play a role in regulating cardiomyocyte cell cycle activity during early postnatal life.

**Results:**

We observed that the expression of ANP and BNP and the level of phosphorylated FOXO were transiently increased in the postnatal mouse heart ventricles, which coincided with the burst of cardiomyocyte cell cycle re-entry during early postnatal life in mice. Cell culture studies showed that ANP/BNP signaling and FOXO cooperatively promoted cell cycle activity in neonatal mouse cardiomyocytes. The enhanced cell cycle activity observed in combined treatment of ANP/BNP and dominant-negative FOXO (DN-FOXO), which can bind FOXO recognition sites on DNA but cannot activate transcription, was primarily mediated through natriuretic peptide receptor 3 (Npr3). In mice, simultaneous application of ANP and DN-FOXO in postnatal hearts reactivated cell cycle in cardiomyocytes, resulting in reduced scar formation after experimental myocardial infarction.

**Conclusions:**

Our data demonstrate the cooperative effects of natriuretic peptide and DN-FOXO on promoting cardiomyocyte cell cycle activity and mouse cardiac repair and regeneration after injury.

**Supplementary Information:**

The online version contains supplementary material available at 10.1186/s12861-020-00236-y.

## Background

Cardiomyocytes proliferate rapidly during fetal life but lose their ability of proliferation soon after birth. However, before terminal withdrawal from the cell cycle, cardiomyocytes undergo another round of cell cycle activity during early postnatal life in mice [1]. While a transient wave of increased DNA synthesis in cardiomyocyte has been observed in postnatal mouse hearts, the molecular mechanisms describing cardiomyocyte cell cycle re-entry remain poorly understood. Atrial and B-type natriuretic peptides (ANP and BNP) are abundantly expressed in embryonic heart ventricles [2–5]. After birth, the expression of both genes is strongly reduced in the ventricular myocardium [2–5]. ANP and BNP elicit their effects by binding to the natriuretic peptide receptors (NPR). Npr1 and Npr2 are guanylyl cyclase-linked receptors. Loss of Npr1 or Npr2 function in mouse hearts resulted in cardiac hypertrophy [6, 7]. In contrast, Npr3 does not have guanylyl cyclase activity. It has been shown that Npr3 binds and internalizes circulating natriuretic peptides, thus functioning as a clearance receptor of circulating natriuretic peptides [8]. Furthermore, the cytoplasmic domain of Npr3 contains a Gi activator sequence that inhibits adenylyl cyclase activity [9–11]. Studies in zebrafish hearts in vivo and in mouse cardiomyocytes in vitro showed that low levels of natriuretic peptides enhanced cardiomyocyte proliferation via the Npr3-dependent signaling pathway [12]. In contrast, high levels of natriuretic peptides inhibit cardiomyocyte proliferation via the Npr1- and Npr2-dependent PKG signaling pathway [12]. These data suggest the important role of the cardiac natriuretic peptides pathway in modulating cardiomyocyte proliferation.

Forkhead O (FOXO) transcription factors are known as important targets of insulin and growth factor action. They are under negative control by the PI3K-Akt/PKB pathway, which phosphorylates FOXO proteins and renders them transcriptionally inactive by promoting their nuclear exclusion [13–16]. FOXO1 and FOXO3 have been implicated in cardiomyocyte cell cycle control. In the prenatal mouse heart, overexpression of FOXO1 or FOXO3 inhibits myocyte proliferation and induces expression of cell cycle inhibitors p21 (p21^CIP1^) and p27 (p27^KIP1^) [17]. In contrast, cardiac-specific overexpression of dominant-negative FOXO, which can bind FOXO recognition sites on DNA but cannot activate transcription, leads to increased myocyte proliferation and reduces expression of cell cycle inhibitors [17]. Thus, cardiomyocyte proliferation is affected by FOXO transcriptional activity. Despite these important findings, it remains largely unknown whether natriuretic peptides and FOXO cooperatively play a role in regulating cardiomyocyte cell cycle activity during early postnatal life.

In this study, we report the expression of ANP and BNP and the level of phosphorylated FOXO in neonatal mouse heart ventricles during early postnatal life. We assess the effects of ANP/BNP signaling and FOXO transcription factors on cell cycle activity in primary neonatal mouse cardiomyocytes in culture. Treatment of infarcted mouse hearts with the natriuretic peptide and dominant-negative FOXO during early postnatal life provides in vivo evidence that natriuretic peptides and FOXO transcription factors work in an integrated manner to control cardiomyocyte cell cycle activity in early postnatal life.

## Methods

### Mice

All litter sizes of wild-type mice (CD-1® IGS, Charles River Laboratories) were adjusted to 8–10 pups per litter. The sex of newborn mice used in these studies was not determined. Generally, the sample size was chosen to use the least number of animals to achieve statistical significance, and no statistical methods were used to predetermine sample size. Animals were divided into experimental groups based on the type of treatment. We did not use exclusion, randomization, or blinding approaches. All the animal experiments were carried out by the NIH guidelines (Guide for the care and use of laboratory animals). All experimental procedures involving animals in this study were reviewed and approved by the Institutional Animal Care and Use Committee of Temple University Medical Center.

### Neonatal cardiomyocyte isolation and culture

The newborn mice (≤ 5 days of age) were euthanized by decapitation. Cardiomyocytes were collected using the method previously described [18]. In short, mouse cardiomyocytes were isolated by enzymatic disassociation of neonate hearts at postnatal day 1 (P1). Cells were differentially seeded for 2 h to remove fibroblasts. Cardiomyocytes were plated at the density of 1.5 × 10^4^ cells per well on a 96-well plate coated with laminin (10 μg/cm^2^). Twenty-four hours later, the culture medium was replaced with fresh medium (Opti-MEM supplemented with 10% fetal bovine serum, 5% horse serum, and 10 Unit/ml Penicillin-Streptomycin) either with or without ANP (1 μM; Phoenix Pharmaceuticals, Inc.; Catalog NO:005–24), BNP (1 μM; Phoenix Pharmaceuticals, Inc.; Catalog NO:011–23), or adenovirus expressing dominant-negative FOXO (Ad-DN-FOXO) (1500 vp/cell), or Ad-Cre (1500 vp/cell). For lentiviral transduction, the culture medium was replaced with fresh medium containing Npr1 shRNA lentivirus (pLKO.1-Npr1 shRNA), Npr2 shRNA lentivirus (pLKO.1-Npr2 shRNA), Npr3 shRNA lentivirus (pLKO.1-Npr1 shRNA), or scramble shRNA lentivirus using 5 μg/ml polybrene (American Bioanalytical, MA). After 48 h, cardiomyocytes were fixed and processed for immunostaining with the indicated antibodies.

### Lentivirus expression in cardiomyocytes

Murine Npr1 shRNA, Npr2 shRNA, and Npr3 shRNA were synthesized and subcloned into pLKO.1 lentiviral vector (Dharmacon Open Biosystems; Catalog # RHS4080). pLKO.1-scramble shRNA plasmid was used as previously described [19]. Lentiviral particles were prepared by transiently transfecting HEK293T cells with lentiviral vectors (pLKO.1-Npr1 shRNA, pLKO.1-Npr2 shRNA, pLKO.1-Npr3 shRNA, or pLKO.1-scramble shRNA) together with packaging vectors (pMD2-VSVG and psPAX2). Viral supernatant was collected at 48 h post-transfection, concentrated, and applied to cardiomyocytes.

### Adenovirus expression in cardiomyocytes

The dominant-negative FOXO (Δ256) protein contains the entire forkhead DNA binding domain but lacks the transactivation domain, thus inhibiting the transcriptional activity of FOXO1 and FOXO3 [20]. Infection with Ad-Cre virus was used as a control for in vivo experiments.

### Histology

Heart tissues were fixed in 4% formaldehyde and processed for paraffin histology and sectioned using routine procedures. Immunohistochemical staining was performed using the previously described protocol [18, 19]. Primary antibodies are: Ki67 (1:50; Abcam, ab16667), cardiac Troponin T (cTnT, 1:100; Thermo Scientific, MS-295-P1). DAPI was used to counterstain nuclei. Apoptosis was measured using In Situ Cell Death Detection Kit (Roche). Cell proliferation was measured using Click-iT® EdU (5-ethynyl-2′-deoxyuridine) Alexa Fluor® Imaging Kit (Thermo). The slides were imaged and subjected to an independent blinded analysis, using a Zeiss LSM 710 confocal microscope and ImageJ software. Images shown are the representative views of five random fields of the left ventricle from at least four independent samples per group. Quantitation of cell numbers was done using images acquired on confocal microscopy and the ImageJ with the “Cell Counter” plug-in, counting multiple fields from at least 4 independent samples per group and about 1000 cTnT+ cells per sample.

### Myocardial infarction and intramyocardial gene transfer and EdU labeling

The myocardial infarction model was used as previously described [21]. Briefly, P7 mice were anesthetized by hypothermia on ice for 3–5 min. The heart was exposed and the left anterior descending (LAD) coronary artery was ligated by a 7–0 polypropylene suture at the lower level of the left atrial appendage. Immediately after ligation of LAD, adenoviral vectors containing the DN-FOXO or Cre or ANP (600 ng) or DN-FOXO+ANP were injected intramuscularly using a syringe with a 30-gauge needle into the border zone surrounding the infarct (1 × 10^9^ vp). The muscle layer and skin were closed and allowed to recover. For EdU labeling, mice were injected with one dose of EdU 50 mg/kg via i.p. injection and sacrificed after 18 h. Mice were euthanized by inhalation of 100% CO2 followed by cervical dislocation.

### In situ hybridization

In situ hybridization was performed using Digoxigenin (DIG) Labeled In Situ Probe on 8-μm paraffin-embedded tissue sections. To synthesize antisense probes for ANP and BNP, part of ANP and BNP mRNA sequences were cloned into the pCR4-TOPO plasmid using the following primers: ANP forward primer TCAAGAACCTGCTAGACC, ANP reverse Primer CCTAGTCCACTCTGGGCT; BNP forward primer ATGCAGAAGCTGCTGGAGCTG, BNP reverse primer GTTACAGCCCAAACGACTGAC. After deparaffinization and rehydration, tissue slides from 2 (P2), 5(P5), and 7(P7) days old mice were treated with 10 μg/ml of proteinase K and fixed in 4% PFA. Pre-hybridization was performed incubating tissue samples in a solution containing formamide 50%, SSC 5x, 0.1% Tween-20, citric acid 10 mM, Yeast tRNA 50 mg/ml, and Heparin 50 μg/ml in a humidified chamber for 2 h. Hybridization was performed incubating tissue section in Hybridization solution containing the denatured digoxigenin-probe and incubating at 70 °C overnight in a humidified chamber. The day after, samples were incubated in blocking buffer (2% goat serum, 2mgl/ml BSA in PBST) for 1 h and then in anti-DIG antibody conjugated to alkaline phosphatase (1:2000 in blocking buffer) overnight at 4 °C. Detection was performed using the substrate 5-Bromo-4-chloro-3-indolylphosphate/Nitro-blue tetrazolium (BCIP/NBT) in Alkaline Phosphatase Buffer (Tris-HCl pH 8.0100 mM, 100 mM NaCl, MgCl2 50 mM, Tween-20 0.1%). Tissue section images were taken on a Nikon Eclipse Ni using a Nikon digital sight ds-U3 camera.

### shRNA sequence and constructs

To produce a Lentiviral Npr1 shRNA expression construct, a sense (CCGGCCGTGCTTCTTCATA) and anti-sense (TATGAAGAAGCACGGCCGG) sequence were used to generate a short hairpin RNA in PLK1.0 lentiviral construct. To produce a Lentiviral Npr2 shRNA expression construct, a sense (TGCGGTTTGTCAGCTCCGA) and anti-sense (TCGGAGCTGACAAACCGCA) sequence were used to generate a short hairpin RNA in PLK1.0 lentiviral construct. To produce a Lentiviral Npr3 shRNA expression construct, a sense (TCAGTCTTGTGGACCGCGT) and anti-sense (ACGCGGTCCACAAGACTGA) sequence were used to generate a short hairpin RNA in PLK1.0 lentiviral construct.

### Quantitative real-time PCR (qRT-PCR) analysis

Total RNA was isolated using Trizol and was used to generate cDNA using random hexamer primers and SuperScript III RT (Invitrogen). Real-time thermal cycling was performed using the StepOne Plus cycler (Applied Biosystems) with SYBR green master mix. Transcript expression values were generated with the comparative threshold cycle (Delta CT) method by normalizing to the expression of the 18S gene. Sequences of the oligonucleotide primers used for qRT-PCR analysis are available in Table 1.

**Table 1:** qRT-PCR primer sequences used in this study

Gene name	Forward	Reverse
*Anp/Nppa*	GGGTAGGATTGACAGGATTGG	CTCCTTGGCTGTT ATCTTCGG
*Bnp/Nppb*	CTGAAGGTGCTGTCCCAGAT	CCTTGGTCCTTCAAGAGCTG
*18 s*	TCAAGAACGAAAGTCGGAGG	GGACATCTAAGGGCATCAC
*Npr1*	GTGAAACGTGTGAACCGGAA	AGCTCCCACAAATCTGGTCA
*Npr2*	GCTGACCCGGCAAGTTCTGT	ACAATACTCGGTGACAATGCAGAT
*Npr3*	GTTCCAAATGCGATCGAATGT	CCCACAACGATTCCTGTCACT

### Assessment of cardiac and plasma natriuretic peptide

Mouse heart ventricular tissue lysis was obtained from heart ventricles homogenized using a tissue grinder on ice. Mouse plasma was obtained from blood samples by cardiac puncture. ANP and BNP levels of mouse hear ventricular tissue lysis and plasma were measured using a commercially available enzyme-linked immunosorbent assay kit (Catalogue # EIAM-ANP-1, EIAM-BNP-1, RayBiotech Inc.) according to the manufacturer’s directions.

### Western blot analysis

Mouse heart ventricles were homogenized in RIPA buffer containing protease and phosphatase inhibitors. Protein concentrations were determined using the BCA (Bicinchoninic Acid) Protein Assay Reagent Kit (Bio-Rad). Protein samples were separated on polyacrylamide gels (10% NuPAGE Bis-Tris Gel) and transferred to nitrocellulose membrane (Bio-Rad). The membranes were blocked with 5% BSA (Bovine Serum Albumin) and incubated with primary antibody diluted in 5% BSA at 4 °C overnight, followed by washing with TBST (TBS containing 0.1% Tween 20) solution. Membranes were incubated with near-infrared fluorophore-conjugated secondary antibodies diluted in TBST for 1 h at room temperature and followed by TBST washing three times. Target protein bands on the membranes were visualized using the Odyssey imaging system (LI-COR Biosciences). Primary antibodies include phospho-FOXO1 (Ser256)(E1F7T) Rabbit mAb (CST, #84192), phospho-FOXO3 (Ser318/321) (CST, #9465), FOXO1 (C29H4) Rabbit mAb (CST, #2880), and FOXO3 (75D8) Rabbit mAb (CST, #2497).

### Statistical analysis

Data are presented as mean ± standard error of the mean (s.e.m.). Multiple groups were compared by one-way ANOVA followed Tukey or Dunnett’s post hoc test. Student’s t test was used when comparing two experimental groups. Normality test was performed to determine whether parametric versus nonparametric testing is used. *P* values are depicted as follows: * *P* < 0.05; ** *P* < 0.01; *** *P* < 0.001; **** *P* < 0.0001. Results with *P* > 0.05 were considered not significant (*n.s*.). All analyses were performed with GraphPad Prism 7.

## Results

### Cardiomyocyte cell cycle reactivation in early postnatal life

**Fig. 1:**
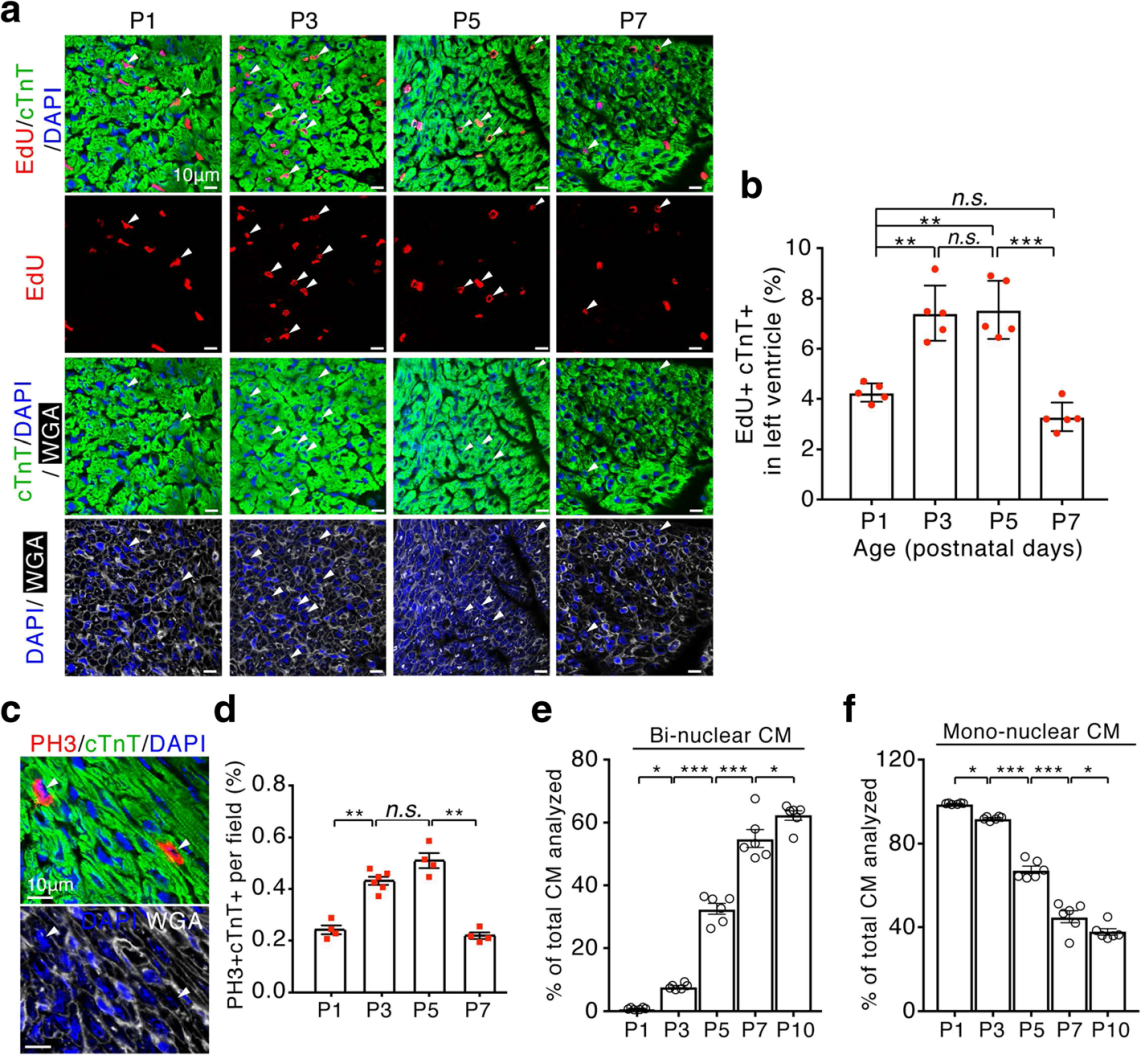
Mouse hearts show evidence for cardiomyocyte cell cycle reactivation during early postnatal life. **a** Cardiomyocytes in the DNA synthesis-phase were visualized by confocal microscope using Click-iT EdU Alexa Fluor (red) and co-immunostaining with antibody against cardiac troponin T (cTnT, green) in sectioned heart left ventricles at indicated postnatal age. Cardiomyocyte boundaries were visualized using WGA staining (white). Cell nuclear were stained with DAPI (blue). Arrows point to EdU+cTnT+ cells. Scale bars: 10 μm. **b** Quantification of EdU+cTnT+ cells as percentage of total cTnT+ cells (~ 1200 cTnT+ cells per sample). **c** Confocal image showing cardiomyocytes in mitotic phase detected by co-immunostainings for phosphorylated histone H3 (PH3, red) and cTnT (green) on heart left ventricular tissue sections. **d** Quantification of PH3+cTnT+ cells as a percentage of total cTnT+ cells analyzed per field. Arrows point to PH3+cTnT+ cells. **e** and **f** Percentage of bi-nuclear **e** and mono-nuclear **f** cardiomyocytes (CM) in the heart ventricles of neonatal mice (*n* = 6 to 8 for all analyzed time points). Data are mean ± s.e.m. *P* value was calculated using Student’s t test. *P < 0.05, **P < 0.01, ***P < 0.001. *n.s*.=not significant

To determine DNA synthesis in cardiomyocytes, neonatal mice received a single intraperitoneal injection of 5-ethyl-2′-deoxyuridine (EdU) and were sacrificed after a 3-h labeling period. The frequency of EdU incorporation was determined on sectioned hearts by co-labeling with the antibody against cardiac troponin T (cTnT) (Fig. 1a). Consistent with previous reports [1, 18], a transient increase of DNA synthesis was observed in neonatal cardiomyocytes, with a peak labeling index occurring between P3 and P5 (Fig. 1b). However, there was a significant drop in the level of EdU+cTnT+ in P7 hearts. The wave of cardiomyocyte cell cycle withdrawal was also observed by immunostaining of sectioned hearts for mitotic cell cycle marker phosphorylated histone H3 (PH3) (Fig. 1c). The levels of PH3+cTnT+ were highest in P3-P5 hearts, followed by a significant drop in P7 hearts (Fig. 1d). To determine whether cell division changes during postnatal life, we quantified the percentage of mono-, and bi-nuclear cardiomyocytes in the ventricles at these time points. The most striking change was the increase in binucleated cardiomyocytes by 4.5 ± 0.01-fold that occurred from P3 to P5, followed by a further increase of binucleated cardiomyocytes by 1.8 ± 0.03-fold from P5 to P7 (Fig. 1e). In contrast, 19.7 ± 0.1% of mononucleated cardiomyocytes were lost from P3 to P5. This was followed by a further loss of 28.6 ± 0.7% mononucleated cardiomyocytes between P5 and P7 (Fig. 1f). These data support the previous observations showing that the wave of postnatal cardiomyocyte cell cycle reactivation, marked by increased DNA synthesis, terminates before cell division, and generates cardiomyocyte binucleation [1, 18].

### A temporal natriuretic peptide signal in mouse heart during early postnatal life

**Fig. 2:**
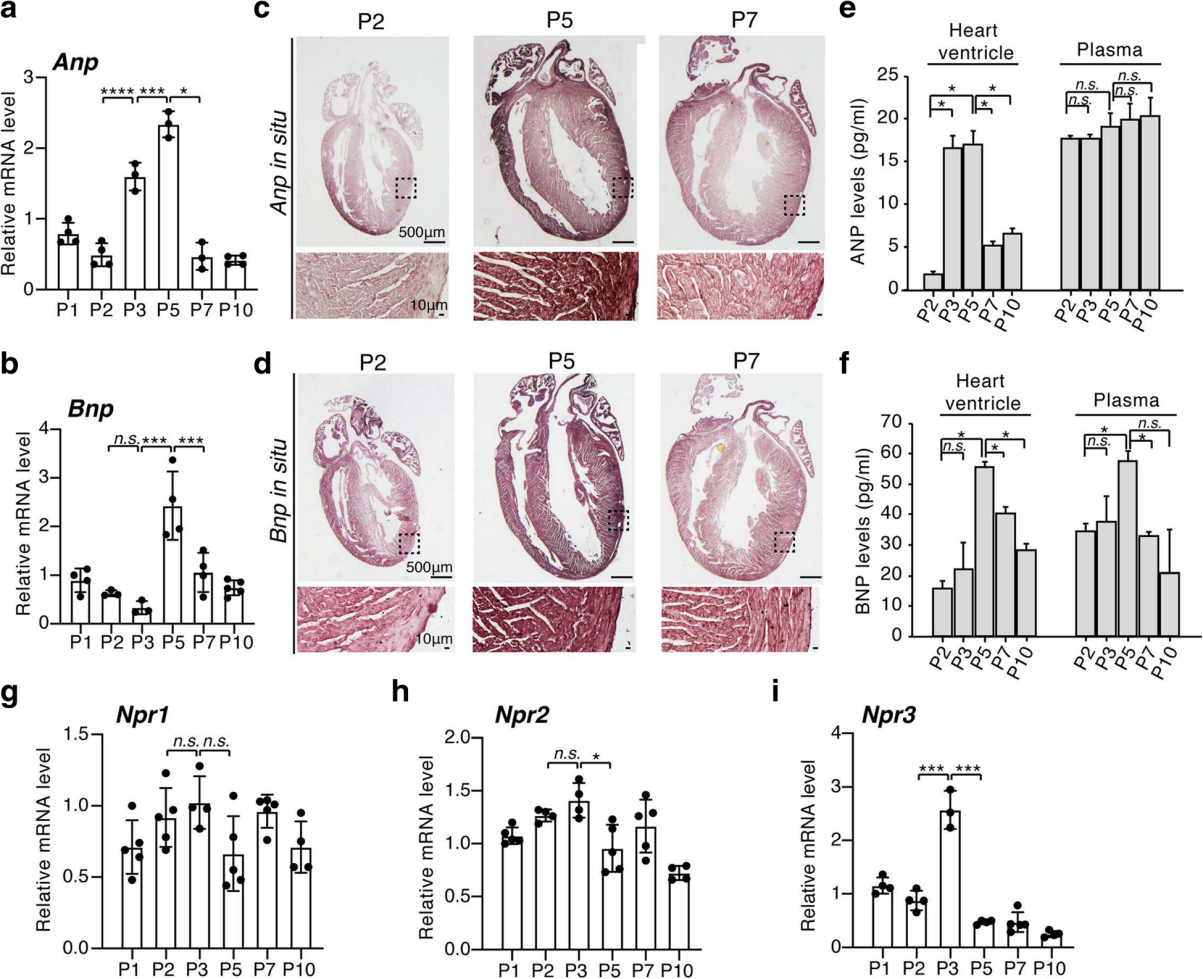
A temporal natriuretic peptide signal in mouse hearts during early postnatal life. **a** and **b** qRT-PCR analysis showing mRNA levels of *Anp* (**a**) and *Bnp* (**b**) in neonatal heart ventricles at indicated time points. **c** and **d** Expression of *Anp* (**c**) and *Bnp* (**d**) was visualized in ventricular myocardial tissues using in situ hybridization. Scale bars: 500 μm (upper images), 10 μm (lower images). **e** and **f** Protein levels of ANP (**e**) and BNP (**f**) in heart ventricles and blood serum (plasma) using ELISA analysis. **g-i** qRT-PCR analysis showing mRNA levels of *Npr1* (**g**), *Npr2* (**h**), and *Npr3* (**i**) in neonatal heart ventricles at indicated time points. *P* value was calculated using one-way ANOVA. *P < 0.05; ***P < 0.001; ****P < 0.0001; *n.s.*= Not significant

**Fig. 3:**
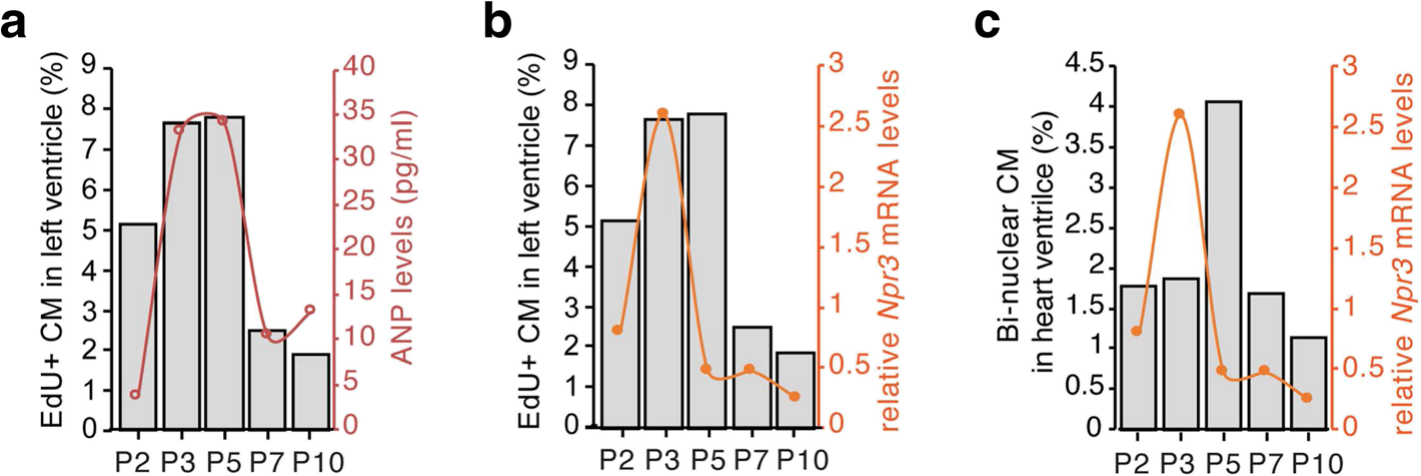
Association of natriuretic peptide levels and cardiomyocyte cell cycle activity in mouse hearts during early postnatal life. **a** and **b** Correlation of transient up-regulation of ANP protein (**a**) and *Npr3* mRNA (**b**) levels in the heart ventricle with the rapid increase in cardiomyocyte cell cycle activity (EdU+ CM) in P3 hearts. **c** Correlation of down-regulation of *Npr3* expression in P5 heart ventricles with the rapid increase in cardiomyocyte bi-nucleation (bi-nuclear CM)

Natriuretic peptide signaling induced by ANP (also known as *Nppa*) and BNP (also known as *Nppb*) are important modulators during cardiomyocyte hypertrophy in adulthood [22, 23]. However, their role in cardiac growth during early postnatal life is largely unknown. We examined the expression of ANP and BNP in neonatal heart ventricles at both mRNA level and protein level. The qRT-PCR analysis showed that ventricular *Anp* mRNA increased by 3 and 4.5-fold on P3 and P5, respectively, compared to P2 (Fig. 2a). *Bnp* mRNA increased by 3.5-fold on P5 compared to P2 (Fig. 2b). Their expression returned to the basal line on P7 (Fig. 2a and b). Increased expression of natriuretic factors in the P5 left ventricular myocardium was also found by in situ hybridization for *Anp* and *Bnp* (Fig. 2c and d). In addition, analysis of ANP protein level by ELISA showed its significant increase in heart ventricles at P3 and P5 compared to P2 and P7 (Fig. 2e). In contrast, the plasma ANP protein level was not significantly changed among all the time points examined (Fig. 2e). Furthermore, BNP protein level was significantly higher in both heart ventricles and plasma at P5 compared to P2 and P7 (Fig. 2f). In contrast to *Anp* and *Bnp*, the expression of natriuretic peptide receptors, *Npr1* and *Npr2,* did not increase as dramatically through the early stages of postnatal life (Fig. 2g and h), and instead *Npr2* expression decreased significantly on P5 compared to P3 (Fig. 2h). However, *Npr3* expression increased significantly on P3, coinciding with peak expression of *Anp* in postnatal heart development (Fig. 2i). Notably, this transient up-regulation of ANP and *Npr3* in heart ventricles coincided with the rapid increase in cardiomyocyte cell cycle activity on P3 hearts (Fig. 3a and b). In contrast, down-regulation of *Npr3* expression in P5 heart ventricles coincided with the rapid increase in cell cycle exit and cardiomyocyte bi-nucleation (Fig. 3c). These data suggested that postnatal cardiomyocytes exhibited a transient increase in natriuretic peptide signal and *Npr3* expression that coincided with cardiomyocyte cell cycle re-entry in mouse hearts during early postnatal life.

### FOXO phosphorylation is transiently elevated in neonatal mouse heart ventricles

**Fig. 4:**
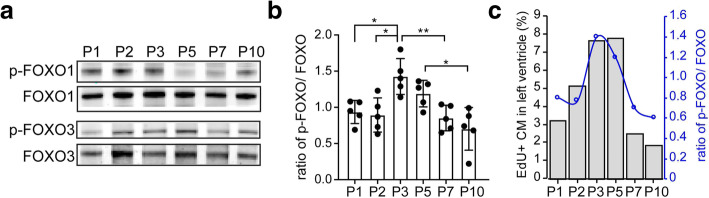
Transient elevation of FOXO phosphorylation in neonatal mouse heart ventricles. **a** Western blot showing levels of FOXO1 and FOXO3 proteins, phosphorylated FOXO1 (p-FOXO1- Ser 256), and phosphorylated FOXO3 (p-FOXO3- Ser 318/321) in the tissue lysates from P1 to P10 heart ventricles. **b** Quantification of the ratio of p-FOXO1/p-FOXO3 to total FOXO1/FOXO3 (p-FOXO/FOXO) in heart ventricles. **c** Correlation of transient elevation of FOXO phosphorylation in heart ventricles with the rapid increase in cardiomyocyte cell cycle activity (EdU+ CM) in P3 hearts. P value was calculated using one-way ANOVA. *P < 0.05; **P < 0.01

FOXO1 and FOXO3 are expressed in developing and adult cardiomyocytes [17]. They are known for cell proliferation in developing hearts and their expression is reduced when the heart reaches the adult stage [7]. FOXO transcriptional activity negatively affects cardiomyocyte proliferation [17]. Upon phosphorylation, FOXO is translocated to the cytoplasm and is transcriptionally inactive [8]. To determine the expression patterns of FOXO transcription factors in the neonatal mouse heart, the protein expression of FOXO family members was examined at various stages of early postnatal life. Both FOXO1 and FOXO3 proteins were detected by western blot in the tissue lysates from P1 to P10 heart ventricles (Fig. 4a and Supplementary Fig. 1). The expression of phosphorylated FOXO1 (p-FOXO1- Ser 256) and phosphorylated FOXO3 (p-FOXO3- Ser 318/321) were also examined and compared with total FOXO1 and FOXO3 respectively. The phosphorylation of FOXO1 and FOXO3 was significantly increased in the heart at P3 compared to P1 and P2, as evidenced by the increased ratio of p-FOXO1/p-FOXO3 to total FOXO1/FOXO3 (thereafter p-FOXO/FOXO) (Fig. 4b). The ratio of p-FOXO/FOXO returned to baseline in P7 and P10 hearts (Fig. 4b). The relative increase in phosphorylated FOXO at P3 suggests decreased nuclear localization and transcriptional activity. Interestingly, this transient up-regulation of phosphorylated FOXO in the heart ventricle coincided with the rapid increase in cardiomyocyte cell cycle activity on P3 hearts (Fig. 4c). These data suggested that postnatal cardiomyocytes exhibited a transient decrease in FOXO nuclear activity that coincided with cardiomyocyte cell cycle reactivation in mouse hearts during early postnatal life.

### ANP/BNP and FOXO cooperatively promote cell cycle activity in neonatal mouse cardiomyocytes in vitro

**Fig. 5:**
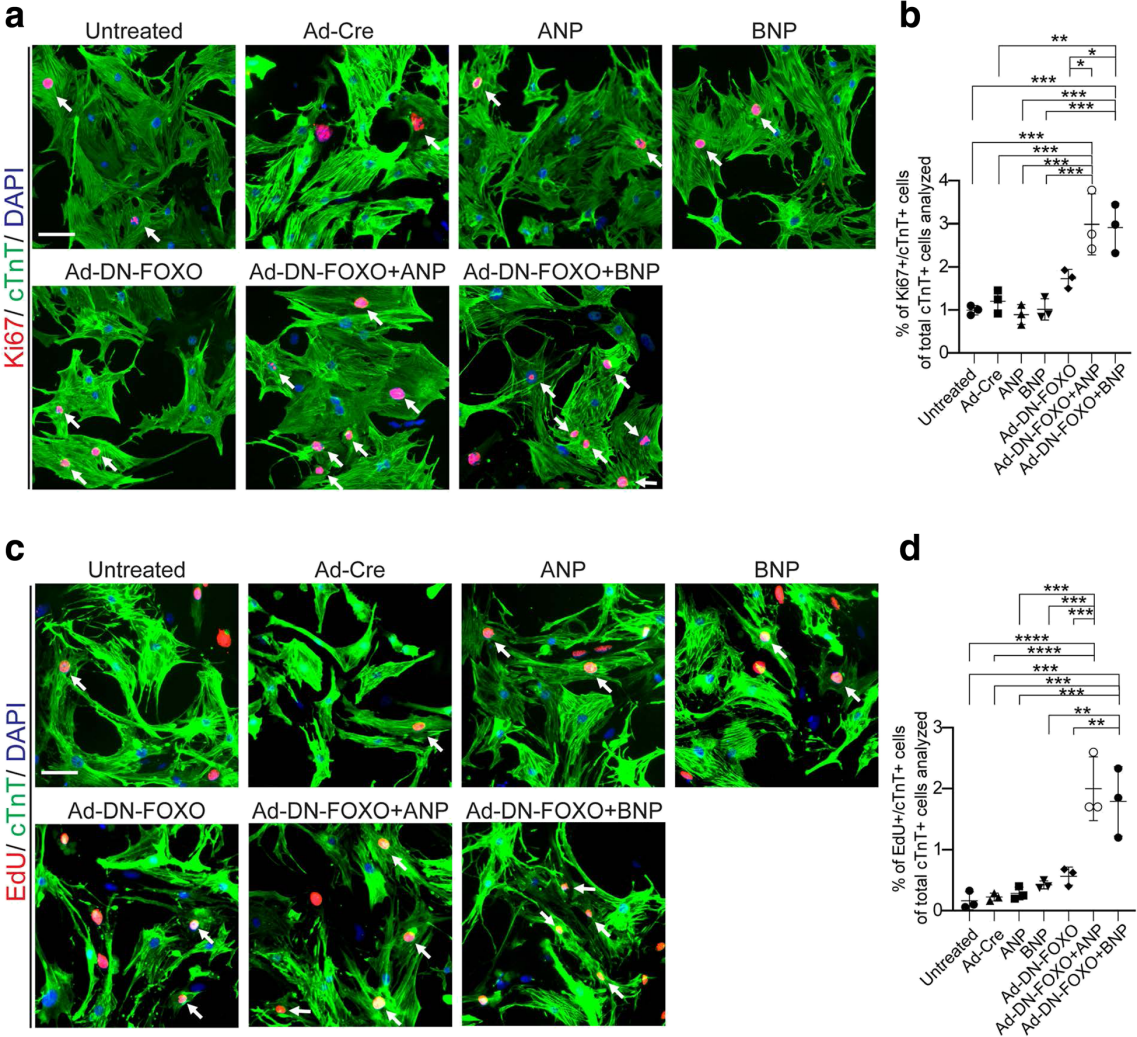
Cardiomyocyte cell cycle activity with natriuretic peptide and DN-FOXO treatment in vitro. **a** Isolated neonatal mouse cardiomyocytes (P1) were cultured with a medium containing Ad-Cre, ANP, BNP, or Ad-DN-FOXO for 48 h. Cardiomyocyte cell cycle activity was visualized by co-immunostaining for Ki67 (red) and cTnT (green) on cultured cardiomyocytes. Arrows point to Ki67+cTnT+ cells. **b** Quantification of Ki67+cTnT+ cells as a percentage of total cTnT+ cells analyzed per field (~ 1000 cTnT+ cells per sample). **c** Cardiomyocytes in the DNA synthesis-phase were detected by using Click-iT EdU Alexa Fluor (red) and co-immunostaining with the antibody against cTnT (green) on tissue sections. Arrows point to EdU+cTnT+ cells. **d** Quantification of EdU+cTnT+ cells as a percentage of total cTnT+ cells analyzed per field (~ 1000 cTnT+ cells per sample). Scale bars: 50 μm. Data are mean ± s.e.m. *P* value was calculated using one-way ANOVA. *P < 0.05; **P < 0.01; ***P < 0.001; ****P < 0.0001; *n.s*.= Not significant

To determine the contribution of natriuretic peptides and FOXO to cardiomyocyte cell cycle activity, neonatal mouse cardiomyocytes were administered with ANP or BNP or infected with adenovirus expressing dominant-negative FOXO (Ad-DN-FOXO), which inhibits transcriptional activity of FOXOs for 48 h. Cardiomyocyte cell cycle activity was evaluated by quantifying cell cycle marker (Ki67) expression and the frequency of EdU incorporation, the indicator of cells in the DNA synthetic phase. These studies showed that administration of either ANP, BNP, or DN-FOXO or Cre alone, had no significant impact on cardiomyocyte cell cycle activity compared to the control cultures (Fig. 5a-d). Notably, combined treatment of ANP/BNP and DN-FOXO resulted in significant up-regulation in cardiomyocytes cell cycle activation index, as evidenced by increases in the percentage of Ki67 and cTnT (cardiomyocyte marker) double-positive cells (Ki67+/cTnT+) as well as EdU and cTnT double-positive cells (EdU+/cTnT+) (Fig. 5a-d). These data show that expression of DN-FOXO and ANP/BNP cooperatively promoted cardiomyocyte cell cycle activity in culture.

### Natriuretic peptide receptor 3 (Npr3) is involved in ANP/BNP and FOXO-mediated increase in cardiomyocyte cell cycle activity

**Fig. 6:**
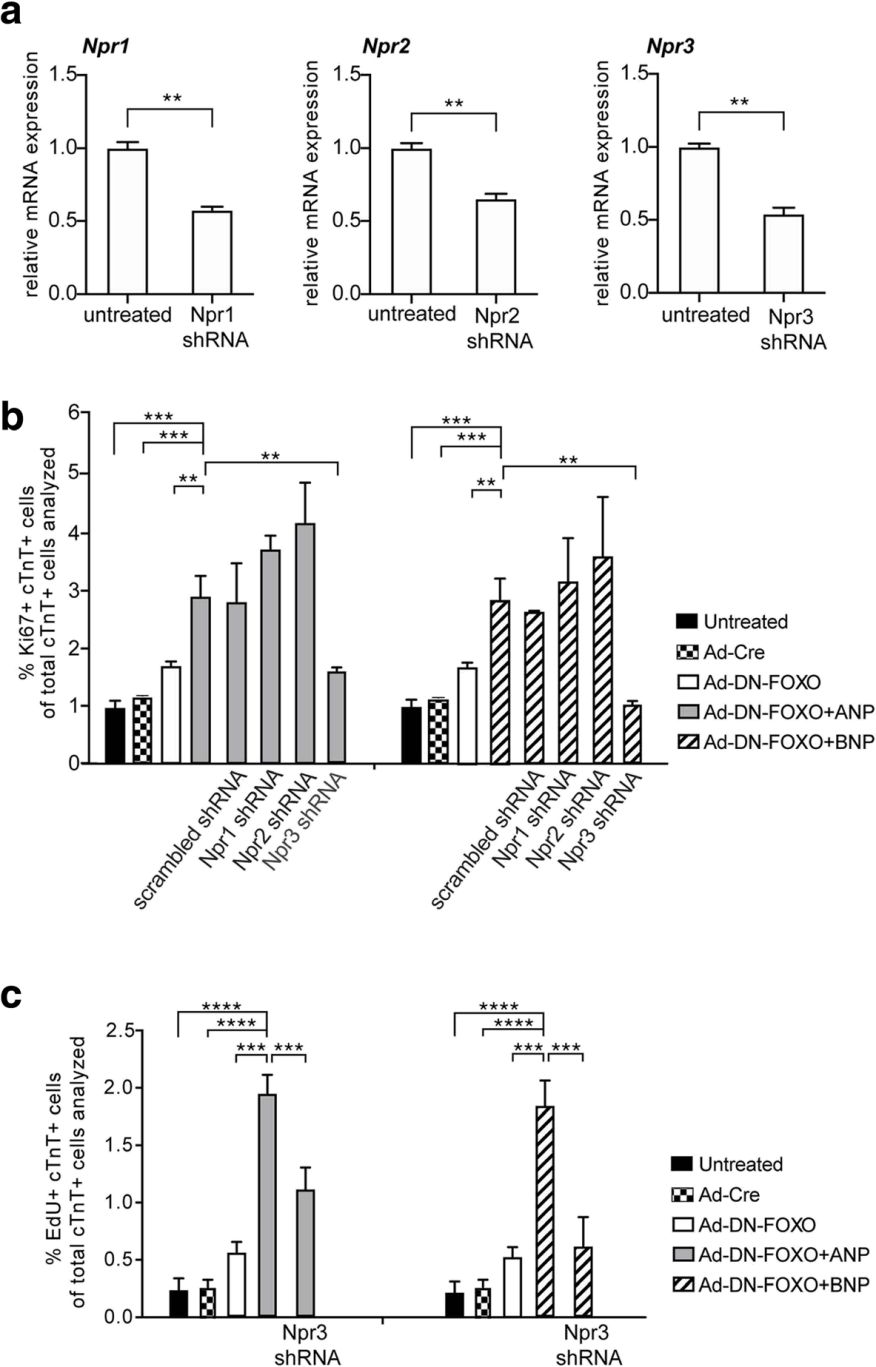
Enhanced cardiomyocyte cell cycle activity observed in the simultaneous treatment of natriuretic peptide and DN-FOXO was primarily mediated through Npr3. **a** qRT-PCR analysis showing expression of *Npr1*, *Npr2,* and *Npr3* in cultured neonatal mouse cardiomyocytes at 48 h after infection with lentivirus expressing either Npr1 shRNA, Npr2 shRNA, or Npr3 shRNA. **b** and **c** Npr3 shRNA treatment abrogating the effects of ANP/BNP and DN-FOXO effects on cardiomyocyte cell cycle activity. Quantification of cardiomyocyte cell cycle activity as the percentage of Ki67+cTnT+ (**b**) and EdU+cTnT+ cells **(c)** of total cTnT+ cells analyzed. *n*=4–5 per group. *P* value was calculated using one-way ANOVA. **P < 0.01; ***P < 0.001; ****P < 0.0001; *n.s*.= Not significant

Natriuretic peptides bind to three natriuretic peptide receptors (Npr1, Npr2, and Npr3) [9]. We investigated if the knockdown of these receptors could reverse the cooperative effects of ANP/BNP and FOXO on cardiomyocyte cell cycle activity. We designed shRNA to disrupt the expression of these genes to generate a loss of function in cardiomyocytes (Fig. 6a). When Npr1 or Npr2 alone were targeted, there were no significant changes in cardiomyocyte cell cycle activity (Fig. 6b). However, inhibiting Npr3 diminished the ANP/BNP and DN-FOXO medicated increase in cardiomyocyte cell cycle activity (Fig. 6b and c). These data indicate that the enhanced cell cycle activity observed in the combined treatment of natriuretic peptide and DN-FOXO was primarily mediated through Npr3.

### ANP and DN-FOXO cooperatively promote cardiomyocyte cell cycle activation and cardiac regeneration after myocardial infarction in neonatal mouse hearts

**Fig. 7:**
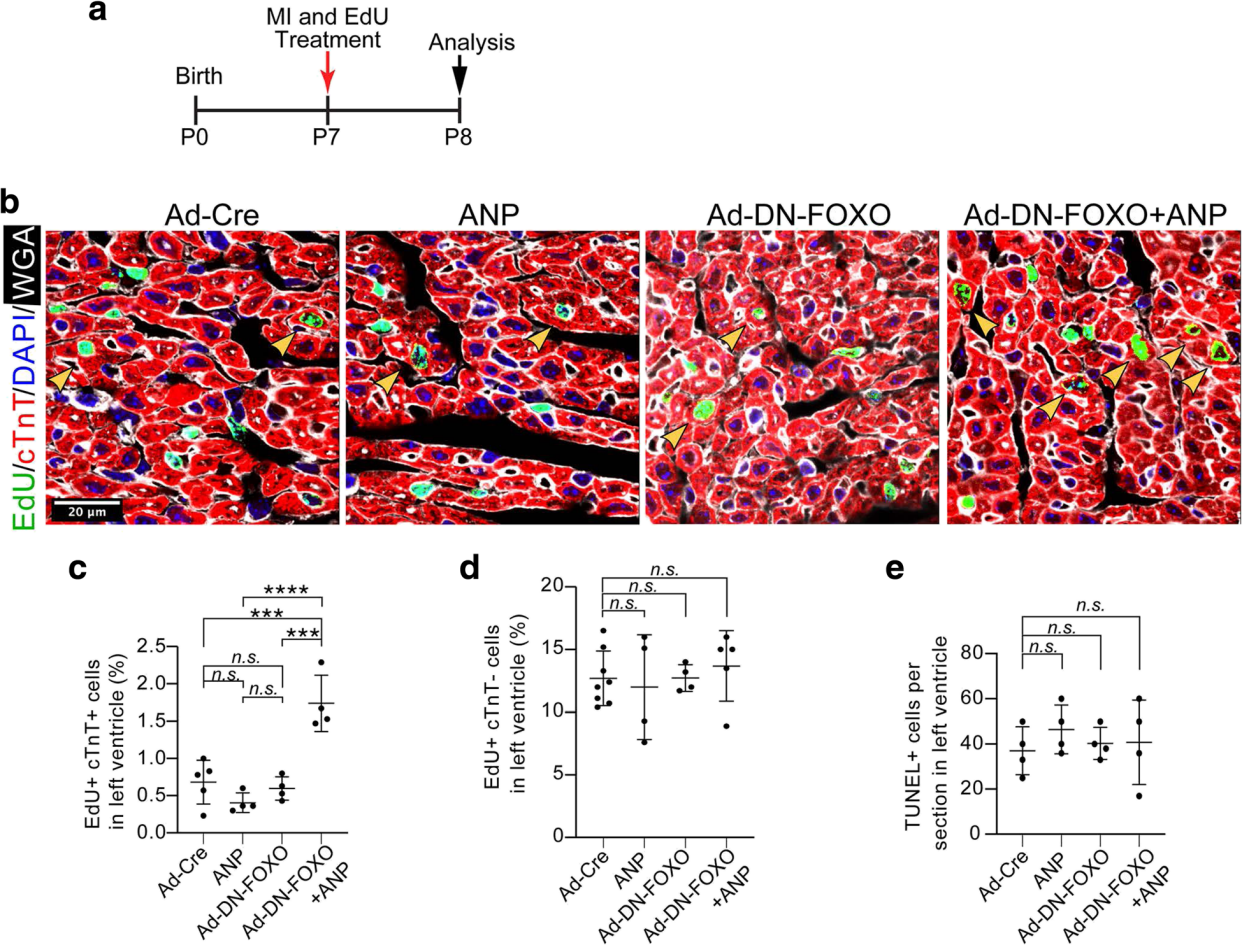
Cardiomyocyte cell cycle activity with ANP and DN-FOXO treatment in vivo. **a** Study design of ANP and DN-FOXO treatment by intramyocardial injection after MI by ligation of the left anterior descending coronary artery. **b** Confocal images showing cardiomyocytes in DNA synthesis-phase visualized using Click-iT EdU Alexa Fluor (green) and co-immunostaining with the antibody against cTnT (red) in sectioned heart ventricles at P8. Arrows point to EdU+cTnT+ cells. Cardiomyocyte boundaries were visualized using WGA staining (white). Scale bars: 20 μm. **c** Quantification of EdU+cTnT+ cells as a percentage of total cTnT+ cells analyzed on heart ventricular sections from P8 hearts (*n*=3–5 per group, ~ 1000 cTnT+ cells per sample). **d** Quantification of EdU+cTnT- cells as a percentage of total cTnT- cells analyzed on heart ventricular sections from P8 hearts. **e** Quantification of cell apoptosis as the number of TUNEL+ cells on heart ventricular sections per field. *P* value was calculated using one-way ANOVA. ***P < 0.001; ****P < 0.0001; *n.s.* = Not significant

Mammalian mouse heart at P7 loses regenerative capacity and develops fibrotic scarring after myocardial infarction (MI) [24]. To determine whether natriuretic peptide and DN-FOXO treatment had an impact on cardiomyocyte cell cycle activity during cardiac repair following injury, CD1 mice on P7 were exposed to MI followed by immediate treatment with ANP and/or Ad-DN-FOXO or Ad-Cre injection into the heart. Heart ventricles were analyzed 24 h after MI. At 18 h before harvesting the heart, EdU was administrated via intraperitoneal injection 6 h after MI (Fig. 7a). Cardiomyocytes within the cell cycle were quantified by visualizing EdU labeled cells co-immunostained with cardiomyocyte marker (cTnT) in heart ventricular tissue sections. These studies showed that administration of Ad-DN-FOXO or ANP alone had no significant impact on cardiomyocyte cell cycle activity compared to the control hearts with Ad-Cre treatment (Fig. 7b and c). However, there was a significant increase in cardiomyocyte cell cycle activity detected in simultaneous treatment of Ad-DN-FOXO and ANP compared to Ad-Cre- or Ad-DN-FOXO- or ANP- treated mice hearts (Fig. 7b and c). In contrast, the cell cycle activity of non-cardiomyocyte was not significantly affected (Fig. 7d). FOXO nuclear activity has been associated with increased apoptosis [25]. We examined cell apoptosis in heart ventricles by TUNEL assay. No significant differences were observed in the simultaneous treatment of Ad-DN-FOXO and ANP compared to Ad-Cre- or Ad-DN-FOXO- or ANP- treated mice hearts (Fig. 7e).

**Fig. 8:**
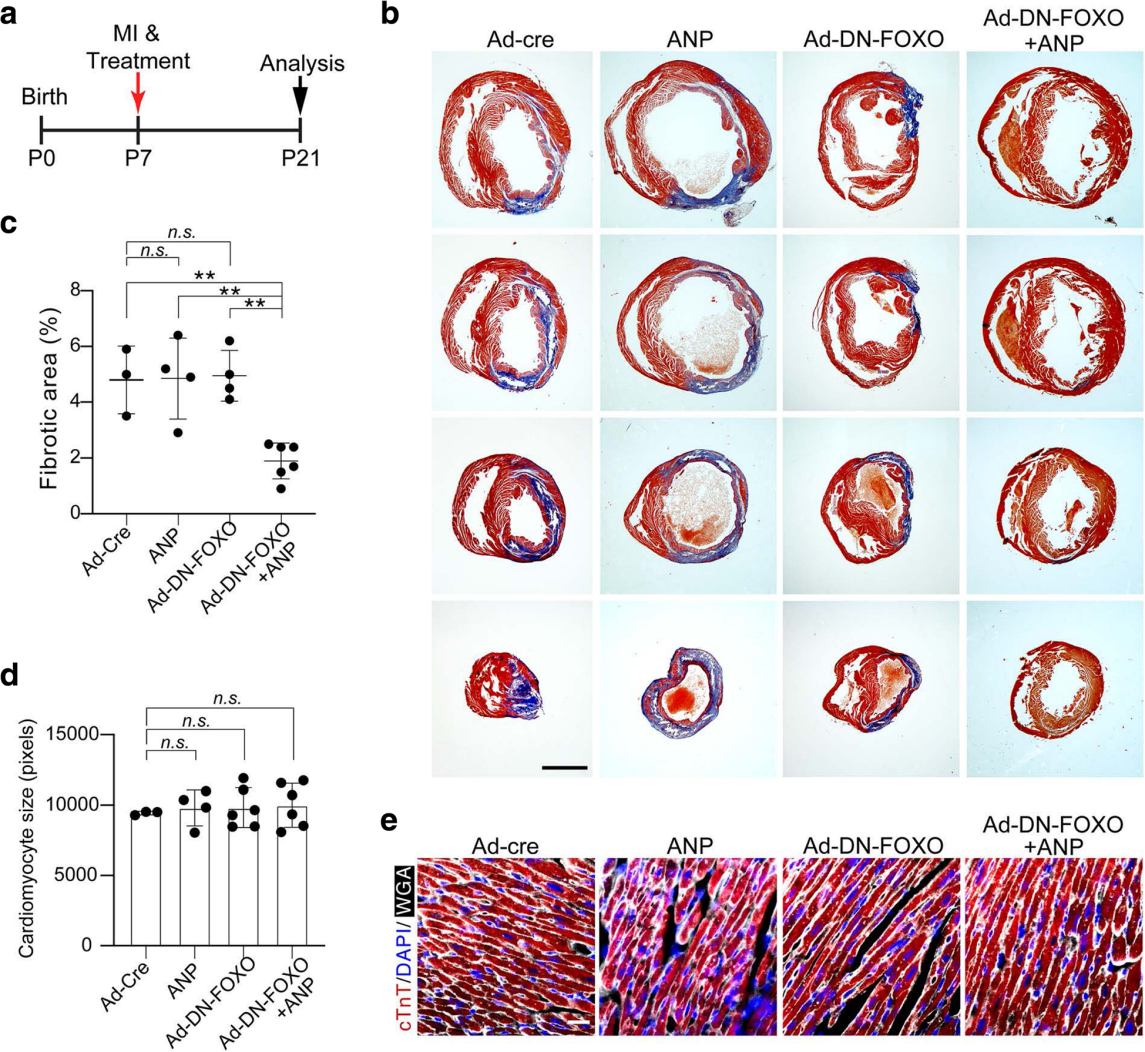
Cardiac fibrosis in ANP and DN-FOXO treated hearts following MI-induced cardiac injury. **a** Schematic of ANP and DN-FOXO treatment after MI. **b** Cardiac fibrotic tissues were visualized using Masson’s trichrome stain on heart ventricular sections 14 days after MI. Serial sections were cut at 500-μm intervals from the size of the ligature toward the apex. Scale bars: 2000 μm. **c** Quantification of fibrotic areas in heart sections. **d** Quantification of relative cardiomyocyte size calculated as cell area from heart section images of co-immunostaining for cTnT and WGA using Image J software. **e** Cardiomyocyte boundaries were visualized using WGA staining (white) and co-immunostaining with the antibody against cardiac troponin T (cTnT, red) in sectioned heart ventricles. Cell nuclear were stained with DAPI (blue). *P* value was calculated using one-way ANOVA. **P < 0.01; *n.s.* = Not significant

To determine whether natriuretic peptides and Ad-DN-FOXO treatment affected cardiac fibrosis formation over the long term, we examined mouse hearts 14 days after MI (Fig. 8a). Analysis of ANP and Ad-DN-FOXO-treated hearts showed that they had significantly less fibrotic scarring than the Ad-Cre- or Ad-DN-FOXO- or ANP- treated hearts (Fig. 8b and c). Furthermore, we examined cardiomyocyte cell size in heart ventricles and found no significant differences in simultaneous treatment of Ad-DN-FOXO and ANP compared to Ad-Cre- or Ad-DN-FOXO- or ANP- treated mice (Fig. 8d and e). Collectively, these data indicate that ANP and DN-FOXO cooperatively promoted cardiomyocyte cell cycle activity both in vitro and in vivo. Treatment of ANP and DN-FOXO in postnatal mouse hearts resulted in improved cardiac repair and regeneration after MI.

## Discussion

In this report, we establish a novel role for the cardiac natriuretic peptides and FOXO transcription factors in regulating cardiomyocytes cell cycle activity during early postnatal life in mice. Our data demonstrate that natriuretic peptides and FOXO transcription factors modulate cardiomyocyte cell cycle activity in a coordinated manner both in culture and in vivo. Specifically, combined treatment with ANP/BNP and DN-FOXO increased cardiomyocyte cell cycle activity, whereas single treatment of ANP/BNP or DN-FOXO did not significantly affect cell cycle activity compared with control cultures (Fig. 5). The enhanced cardiomyocyte cell cycle activity observed in the combined treatment of natriuretic peptide and DN-FOXO was primarily mediated through Npr3 (Fig. 6).

Natriuretic peptides are dynamically expressed during heart development [2, 3, 12]. ANP and BNP are enriched in embryonic heart ventricles. After birth, the expression of both genes is strongly reduced in the ventricular myocardium. FOXOs are expressed in both embryonic and postnatal heart ventricles [17]. Their transcriptional activity negatively affects cardiomyocyte proliferation. Upon phosphorylation, FOXO is translocated to the cytoplasm and is transcriptionally inactive [13–16]. Here, we showed that the expression of ANP and BNP and the level of phosphorylated FOXO were transiently increased in the postnatal mouse heart ventricles, which coincided with the burst of cardiomyocyte cell cycle re-entry during early postnatal life in mice.

Npr1, Npr2, and Npr3 are expressed in cardiomyocytes during mammalian heart development [12]. Both Npr1 and Npr2 are guanylyl cyclase-linked receptors. They act through the cGMP-PKG signaling pathway [9]. Previous studies show that activation of Npr1 and Npr2 inhibits cardiomyocyte proliferation through a cGMP/PKG-mediated pathway [12, 26]. In contrast, Npr3 does not have guanylyl cyclase activity and functions as a clearance receptor by binding and internalizing circulating natriuretic peptides [8]. Additionally, the Gi activator sequence of the cytoplasmic domain of Npr3 suppresses adenylyl cyclase activity [9–11]. Studies in zebrafish hearts in vivo and in mouse cardiomyocytes in vitro showed that low levels of natriuretic peptides enhanced cardiomyocyte proliferation via Npr3-dependent signaling pathway [12]. Our in vitro data showed that Npr3 knockdown in cultured mouse neonatal cardiomyocytes abrogated the effects of combined treatment of natriuretic peptide and DN-FOXO on cardiomyocyte cell cycle activity (Fig. 6). In contrast, either Npr1 or Npr2 knockdown did not affect cardiomyocyte cell cycle activity. These data suggest that the enhanced cell cycle activity observed in the combined treatment of natriuretic peptide and DN-FOXO was primarily mediated through Npr3. The decreased cardiomyocyte cell cycle activity observed in cultured mouse neonatal cardiomyocytes with Npr3 knockdown may be a secondary cause of the elimination of Npr3 functions including Gi activator and natriuretic peptide clearance. Further in vivo studies using cardiomyocyte-specific Npr3 deletion will separate the receptor’s inhibitory effects on adenylyl cyclase from its role as a systemic natriuretic peptide level regulator, thereby helping to determine its role in cardiomyocyte cell cycle activity.

Our study indicates a new approach that delivers a proliferative stimulus to the injured heart using the simultaneous treatment of ANP and DN-FOXO. Although we did not observe any effects in non-cardiomyocytes, such an adenovirus approach of a proliferative stimulus could affect cellular homeostasis over the long term. The target application of ANP and DN-FOXO using bioengineered delivery approach may reduce the potential for off-target effects in the future.

## Conclusions

In summary, the current findings indicate that ANP/BNP signaling and FOXO nuclear activity cooperatively regulated cardiomyocyte cell cycle activity during early postnatal life in mice. Activation of ANP/BNP signaling and inhibition of FOXO nuclear activity promoted cardiomyocyte cell cycle activity in culture, which was primarily mediated through Npr3. Simultaneous application of ANP and DN-FOXO in postnatal hearts reactivated the cell cycle in cardiomyocytes, resulting in reduced scar formation after experimental myocardial infarction. Our data demonstrate the cooperative effects of natriuretic peptide signaling and FOXO nuclear activity on promoting cardiomyocyte cell cycle activity and mouse cardiac repair and regeneration after injury.

## Supplementary Information

**Additional file 1: Supplementary Figure 1.** Unprocessed versions of western blot images used in Fig. 4a. Western blot showing levels of phosphorylated FOXO1 (p-FOXO1- Ser 256) (a), FOXO1 (b), phosphorylated FOXO3 (p-FOXO3- Ser 318/321) (c), and FOXO3 (d) proteins in the tissue lysates from P1 to P10 heart ventricles. Experimental procedure: Protein extracts were analyzed on polyacrylamide gels (10% NuPAGE Bis-Tris Gel) and transferred to nitrocellulose membrane. Based on the size of examined proteins, the blots were cut between 50 kDA and 75 kDA. The blots of 50 kDA-100 kDA were used for incubation with primary antibody overnight at 4 °C. After washing with TBST, the blots were incubated with near-infrared fluorophore-conjugated secondary antibodies for 1 h at room temperature. Signals were detected using the Odyssey imaging system (LI-COR Biosciences).

## Data Availability

The datasets used and/or analyzed during the current study are available from the corresponding author on reasonable request.

## References

[1] Soonpaa MH, Kim KK, Pajak L, Franklin M, Field LJ (1996). Cardiomyocyte DNA synthesis and binucleation during murine development. Am J Phys.

[2] Bloch KD, Seidman JG, Naftilan JD, Fallon JT, Seidman CE (1986). Neonatal atria and ventricles secrete atrial natriuretic factor via tissue-specific secretory pathways. Cell..

[3] Zeller R, Bloch KD, Williams BS, Arceci RJ, Seidman CE (1987). Localized expression of the atrial natriuretic factor gene during cardiac embryogenesis. Genes Dev.

[4] Horsthuis T, Houweling AC, Habets PE (2008). Distinct regulation of developmental and heart disease-induced atrial natriuretic factor expression by two separate distal sequences. Circ Res.

[5] Tanaka M, Chen Z, Bartunkova S, Yamasaki N, Izumo S (1999). The cardiac homeobox gene Csx/Nkx2.5 lies genetically upstream of multiple genes essential for heart development. Development..

[6] Holtwick R, van Eickels M, Skryabin BV (2003). Pressure-independent cardiac hypertrophy in mice with cardiomyocyte-restricted inactivation of the atrial natriuretic peptide receptor guanylyl cyclase-a. J Clin Invest.

[7] Langenickel TH, Buttgereit J, Pagel-Langenickel I (2006). Cardiac hypertrophy in transgenic rats expressing a dominant-negative mutant of the natriuretic peptide receptor B. Proc Natl Acad Sci U S A.

[8] Nussenzveig DR, Lewicki JA, Maack T (1990). Cellular mechanisms of the clearance function of type C receptors of atrial natriuretic factor. J Biol Chem.

[9] Anand-Srivastava MB, Sehl PD, Lowe DG (1996). Cytoplasmic domain of natriuretic peptide receptor-C inhibits adenylyl cyclase. Involvement of a pertussis toxin-sensitive G protein. J Biol Chem.

[10] Lelièvre V, Hu Z, Ioffe Y (2006). Paradoxical antagonism of PACAP receptor signaling by VIP in Xenopus oocytes via the type-C natriuretic peptide receptor. Cell Signal.

[11] Murthy KS, Makhlouf GM (1999). Identification of the G protein-activating domain of the natriuretic peptide clearance receptor (NPR-C). J Biol Chem.

[12] Becker JR, Chatterjee S, Robinson TY (2014). Differential activation of natriuretic peptide receptors modulates cardiomyocyte proliferation during development. Development..

[13] Brunet A, Bonni A, Zigmond MJ (1999). Akt promotes cell survival by phosphorylating and inhibiting a Forkhead transcription factor. Cell..

[14] Kops GJ, de Ruiter ND, De Vries-Smits AM, Powell DR, Bos JL, Burgering BM (1999). Direct control of the Forkhead transcription factor AFX by protein kinase B. Nature..

[15] Rena G, Guo S, Cichy SC, Unterman TG, Cohen P (1999). Phosphorylation of the transcription factor forkhead family member FKHR by protein kinase B. J Biol Chem.

[16] Kops GJ, Medema RH, Glassford J (2002). Control of cell cycle exit and entry by protein kinase B-regulated forkhead transcription factors. Mol Cell Biol.

[17] Evans-Anderson HJ, Alfieri CM, Yutzey KE (2008). Regulation of cardiomyocyte proliferation and myocardial growth during development by FOXO transcription factors. Circ Res.

[18] Cao T, Liccardo D, LaCanna R, et al. Fatty Acid Oxidation Promotes Cardiomyocyte Proliferation Rate but Does Not Change Cardiomyocyte Number in Infant Mice. Front Cell Dev Biol. 2019;7:42. Published 2019 Mar 22. doi:10.3389/fcell.2019.00042. 10.3389/fcell.2019.00042 PMC6440456 30968022

[19] Tian Y, Liu Y, Wang T (2015). A microRNA-hippo pathway that promotes cardiomyocyte proliferation and cardiac regeneration in mice. Sci Transl Med.

[20] Sengupta A, Kalinichenko VV, Yutzey KE (2013). Circ Res.

[21] Mahmoud AI, Porrello ER, Kimura W, Olson EN, Sadek HA (2014). Surgical models for cardiac regeneration in neonatal mice. Nat Protoc.

[22] Abassi Z, Karram T, Ellaham S, Winaver J, Hoffman A (2004). Implications of the natriuretic peptide system in the pathogenesis of heart failure: diagnostic and therapeutic importance. Pharmacol Ther.

[23] Gardner DG (2003). Natriuretic peptides: markers or modulators of cardiac hypertrophy?. Trends Endocrinol Metab.

[24] Lam NT, Sadek HA (2018). Neonatal heart regeneration: comprehensive literature review. Circulation..

[25] Senokuchi T, Liang CP, Seimon TA (2008). Forkhead transcription factors (FoxOs) promote apoptosis of insulin-resistant macrophages during cholesterol-induced endoplasmic reticulum stress. Diabetes..

[26] O'Tierney PF, Chattergoon NN, Louey S, Giraud GD, Thornburg KL (2010). Atrial natriuretic peptide inhibits angiotensin II-stimulated proliferation in fetal cardiomyocytes. J Physiol.

