# Supplementary figures and images for: Natriuretic peptides and Forkhead O transcription factors act in a cooperative manner to promote cardiomyocyte cell cycle re-entry in the postnatal mouse heart
Source: BMC Dev Biol. 2021 Feb 3;21:6. doi: 10.1186/s12861-020-00236-y (PMC7856820; doi:10.1186/s12861-020-00236-y)

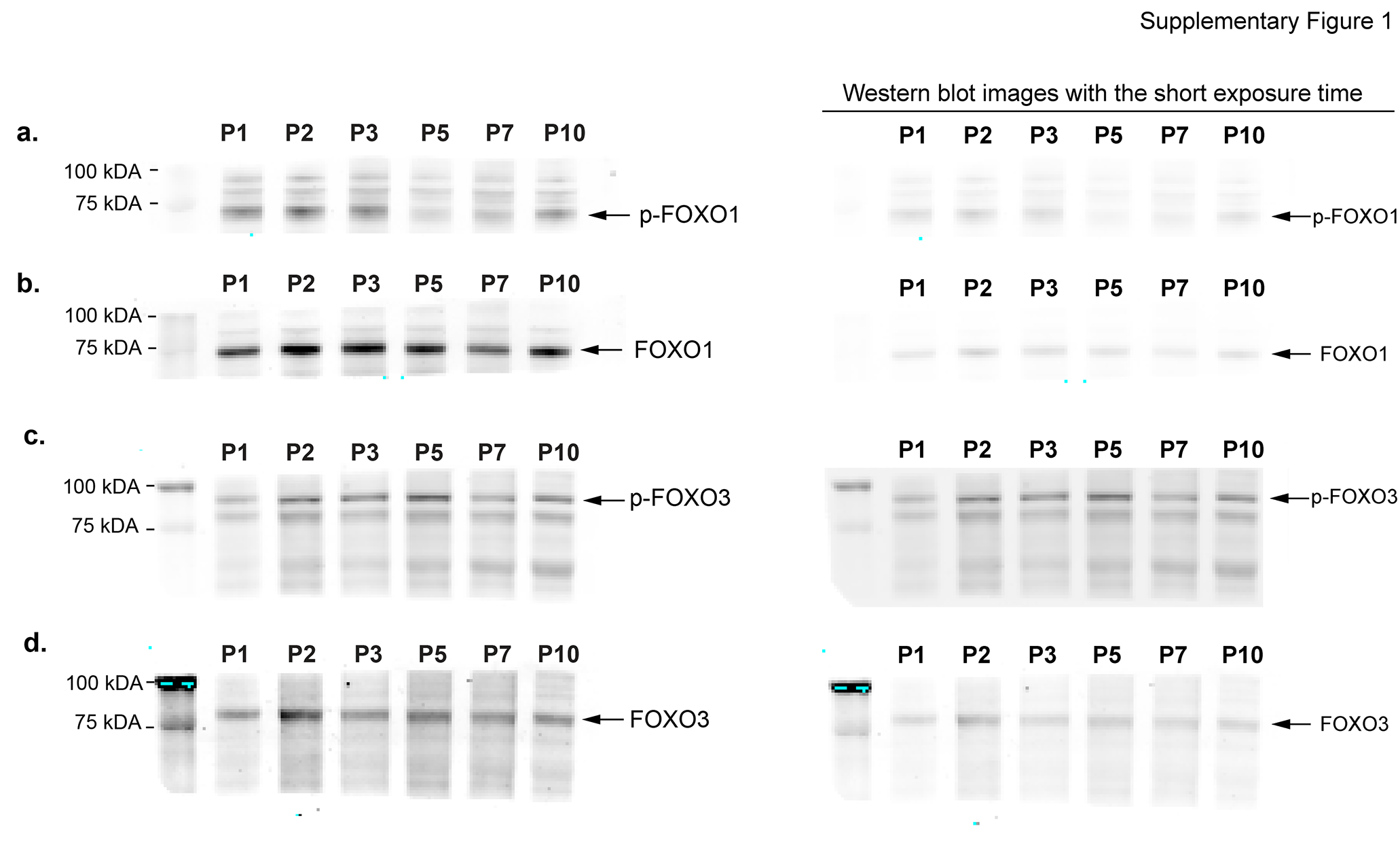

Supplement: Supplementary file 1 — Additional file 1: Supplementary Figure 1. Unprocessed versions of western blot images used in Fig. 4a. Western blot showing levels of phosphorylated FOXO1 (p-FOXO1- Ser 256) (a), FOXO1 (b), phosphorylated FOXO3 (p-FOXO3- Ser 318/321) (c), and FOXO3 (d) proteins in the tissue lysates from P1 to P10 heart ventricles. Experimental procedure: Protein extracts were analyzed on polyacrylamide gels (10% NuPAGE Bis-Tris Gel) and transferred to nitrocellulose membrane. Based on the size of examined proteins, the blots were cut between 50 kDA and 75 kDA. The blots of 50 kDA-100 kDA were used for incubation with primary antibody overnight at 4 °C. After washing with TBST, the blots were incubated with near-infrared fluorophore-conjugated secondary antibodies for 1 h at room temperature. Signals were detected using the Odyssey imaging system (LI-COR Biosciences). [file 12861_2020_236_MOESM1_ESM.tif]
